# Retained Rim Enhancement in Mucinous Breast Cancer Suggests Aggressive Biology: A Case Report

**DOI:** 10.7759/cureus.79644

**Published:** 2025-02-25

**Authors:** Harumasa Takahashi, Shoji Oura

**Affiliations:** 1 Department of Surgery, Kishiwada Tokushukai Hospital, Kishiwada, JPN

**Keywords:** breast cancer, high ki-67 labelling index, mucinous breast cancer, retained rim enhancement, thick fibrous septa

## Abstract

A 44-year-old premenopausal woman was referred to our hospital for a detailed examination of her left rapidly growing breast mass. Mammography only showed focal asymmetric density. Ultrasound showed a 14 mm oval mass with distinct borders, a high depth/width rate of 0.79, numerous punctate echogenic foci against background low echoes, and slightly enhanced posterior echoes. Magnetic resonance imaging (MRI) of the mass showed low signal intensity on T1-weighted images and high signal intensity with a slightly low signal intensity area in the mass center on fat-suppressed T2-weighted images. Subtraction MRI depicted early and retained rim enhancement patterns. The patient underwent a core needle biopsy of the breast mass. Pathological study showed that the tumor had atypical cells growing in a papillary fashion with abundant mucus, leading to the diagnosis of mucinous breast cancer. The patient, therefore, underwent breast-conserving surgery and sentinel node biopsy. A post-operative pathological study showed that the tumor had cancer cells mainly distributed in a ring fashion and extremely sparse cancer cells with thick fibrous septa in the mass center. Immunostaining showed that the mucinous breast cancer had estrogen and progesterone receptor positivities, human epidermal growth factor receptor type 2 equivocation, and a high Ki-labelling index of 50%. Diagnostic physicians should note that retained rim enhancement may predict the biological aggressiveness of mucinous breast cancers.

## Introduction

Mucinous breast cancer is one of breast cancer's special subtypes and is roughly grouped into a mixed subtype, i.e., a phenotype combined with mucinous carcinoma and invasive ductal carcinoma, and a pure subtype. The biology of the former is determined by the co-existing invasive ductal carcinoma components, whereas that of the latter is generally favorable [1]. In short, pure mucinous breast cancer is usually hormone-sensitive, has no lymph node metastasis, and has a low Ki-67 labeling index.

Mucinous breast cancers present characteristic image findings due to the abundant presence of mucus around the cancer cells. Typical images of them are well-demarcated masses with distinct borders, internal high echoes on ultrasound [2], and a plateau or persistent pattern on subtraction magnetic resonance images (MRI) [3]. Internal high echoes are generated by the ultrasound wave backscattering due to the markedly different acoustic impedance between the cancer cells and the surrounding mucus [4,5]. A plateau or a persistent pattern on MRI is caused by the abundant presence of mucus around sparse cancer cells [3].

We herein report a very rare aggressive pure mucinous breast cancer with retained rim enhancement on MRI.

## Case presentation

**Figure 1:**
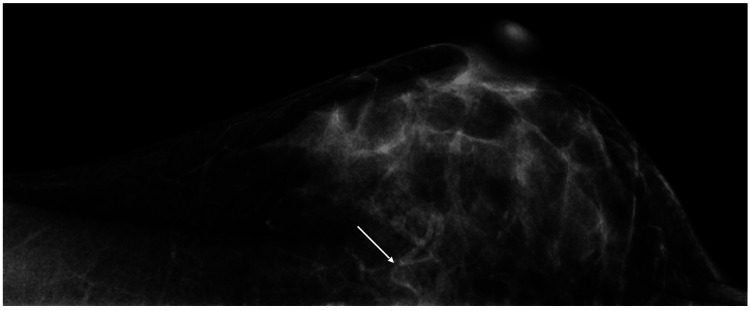
Mammography findings. Mammography only showed focal asymmetric density (arrow).

A 44-year-old woman noticed that a very small nodule in the inner upper quadrant of her left breast had rapidly grown in size over only two months. Mammography only showed focal asymmetric density in the left breast (Figure 1). 

**Figure 2:**
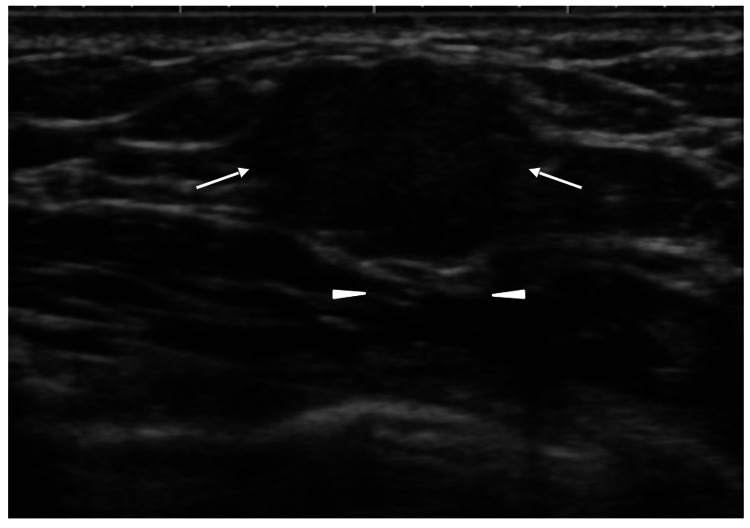
Ultrasound findings. Ultrasound showed an oval mass (arrows) with distinct borders, low internal echoes, punctate echogenic foci throughout the mass, and a faint posterior echo enhancement (arrowheads).

Ultrasound showed a 14 mm oval mass with distinct borders, a high depth/width rate of 0.79, numerous punctate echogenic foci against background low echoes, and slightly enhanced posterior echoes (Figure 2).

**Figure 3:**
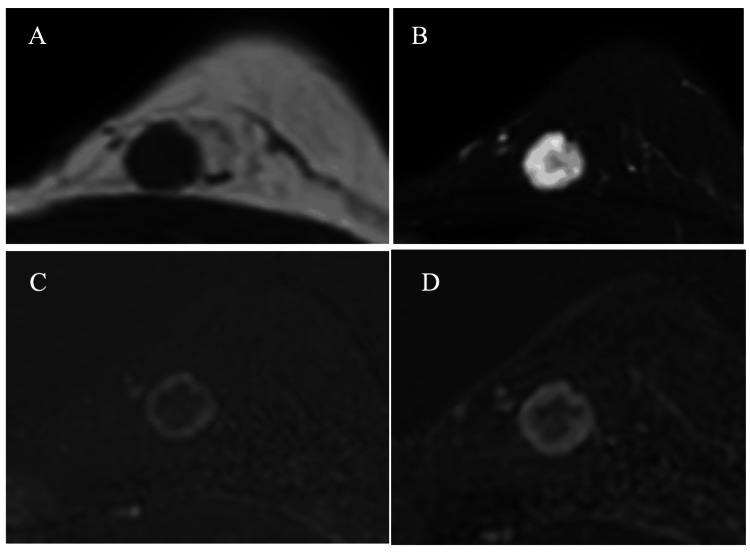
Magnetic resonance image (MRI) findings. MRI of the tumor showed an oval mass basically with low and high signals on (A) T1-weighted images and (B) T2-weighted images, respectively. The center of the tumor had somewhat low signals (B) and long-lasting rim enhancement, (C) early phase, (D) delayed phase on T2-weighted images and subtraction images, respectively.

MRI of the mass showed low signal intensity on T1-weighted images and high signal intensity with a slightly low signal intensity area in the mass center on fat-suppressed T2-weighted images. Subtraction MRI depicted early and retained rim enhancement patterns (Figure 3).

**Figure 4:**
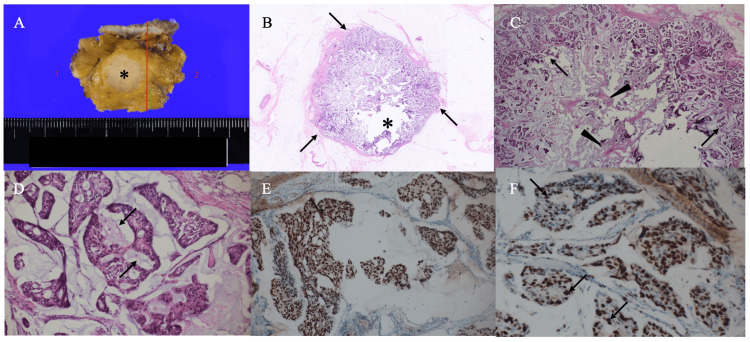
Pathological findings. (A) Bisected tumor showed a whitish oval mass with distinct borders (B) Low magnified view showed a lobulated mass with a presumed artificial defect (asterisk) and thin fibrous components (arrows) surrounding the mass (C) Magnified view showed that the tumor had cancer cells (arrows) in the peripheral areas of the tumor and fibrous septa (arrowheads) with sparse cancer cells in the center of the tumor (D) In the cancer cell clusters, mucin (arrows) was observed in the micro lumens (E) Immunostaining showed estrogen receptor positivity of Allred score 8 (F) Cancer cells forming micro lumens (arrows) were strongly positive for Ki-67 immunostaining.

Under the tentative diagnosis of breast cancer, the patient underwent a core needle biopsy of the breast mass. Pathological study showed that atypical cells grew in a papillary fashion with abundant mucus, leading to the diagnosis of localized early mucinous breast cancer. The patient, therefore, underwent breast-conserving surgery and sentinel node biopsy. A post-operative pathological study showed that the tumor, i.e., pT1, had cancer cells mainly distributed in a ring fashion, extremely sparse cancer cells with thick fibrous septa in the mass center, and no lymph node metastasis (Figures 4A-4C). Immunostaining showed that the mucinous breast cancer had estrogen and progesterone receptor positivities, human epidermal growth factor receptor type 2 equivocation (Hercep test), and a high Ki-labelling index of 50% (Figure 4D). Immunostaining showed estrogen receptor positivity of Allred score 8 and cancer cells forming micro lumens (arrows) were strongly positive for Ki-67 immunostaining (Figures 4E-4F).

The patient was discharged two days after the operation. Despite the explanation of postoperative chemotherapy due to the tumor aggressiveness, the patient chose not to undergo chemotherapy and has been receiving endocrine therapy for six months after adjuvant radiotherapy to the conserved breast.

## Discussion

Mammography generally shows mucinous breast cancers to be high-density masses due to the high X-ray attenuation coefficient of the mucus [6]. In addition, no interminglement of fibrous components and cancer cells at the mass borders generally makes well-demarcated mucinous breast cancers on mammography [7]. In this case, mammography only showed focal asymmetric density due both to the background dense breast and the mass location in the inner upper quadrant of the breast. Mammography, therefore, only suggested the presence of a mass and did not contribute to the image diagnosis of the mass.

Mucinous breast cancers have internal iso or high echoes due to the massive backscattering of ultrasound waves caused by the mechanisms mentioned above [2,7]. Furthermore, both abundant mucus and cancer cell clusters make the posterior echoes of mucinous breast cancers enhanced in the vast majority of cases. This case, however, only had faint posterior echo enhancement, highly suggesting the presence of some intra-mass components that attenuate ultrasound waves.

Abundant mucus with sparse cancer cells makes the pure mucinous breast cancers show high signal intensity on fat-suppressed T2-weighted images and a plateau or a persistent pattern on subtraction MRI [3]. This case, however, had a slightly low signal intensity area in the mass center on fat-suppressed T2-weighted images, suggesting the presence of some components with fewer protons. Retained rim enhancement up to the late phase further suggested the absence or very spare presence of cancer cells in the mass center.

Fibroblast growth factors (FGFs) not only induce fibrosis but also have been shown to activate signal pathways such as RAS/MAPK and PI3 kinase/AKT pathways, thereby promoting the proliferation of tumor cells [8,9]. In particular, the PI3K-Akt pathway is known to be involved in fundamental cellular processes including protein synthesis, proliferation, and survival, and is likely to correlate with the aggressiveness of tumors. In this case, a large amount of both thick fibrous septa and mucus was observed in the mass center, while only very few cancer cells were observed in that area. Mucinous breast cancers often have thin fibrous septa but rarely have thick fibrous components. which might indicate the high proliferation ability of the tumor. Therefore, the presence of thick fibrous septa seen in this case may indicate that the tumor has the ability to produce FGFs, in other words, the tumor has a high proliferation ability [8,9]. Retained rim enhancement, therefore, indirectly suggests the presence of a large number of fibrous components in the mass center and may suggest that mucinous breast cancers with such an imaging finding have aggressive biology.

Although the lack of some mass parts of the tumor probably due to slide preparation procedures, often seen in papillary lesions, made accurate pathological assessment difficult in this case, pathological study showed the presence of thick fibrous septa in the central part of the mass. The thick fibrous septa had only a small number of cancer cells around them. These thick fibrous septa made the posterior echoes somewhat attenuated and T2-weighted images of the mass center were slightly hypo-intense. In addition, retained rim enhancement is well-matched to the very sparse cancer cells in the mass center.

Both ER and PgR positivities on immunostaining seemed to be ordinary findings in mucinous breast cancers [10]. It, however, is noteworthy that this case had a very high Ki-67 labeling index of 50%. The international Ki67 in breast cancer working group recommended that a KI-67 labeling index of 30% or more can be used to estimate prognosis [11]. Therefore, the Ki-67 labeling index of 50% in this case strongly indicates the aggressiveness of breast cancer. Hematoxylin and eosin pathological images clearly showed that mucus was secreted into the micro-lumens in the cribriform structures of the cancer cell clusters. Cancer cells encompassing the micro-lumens were highly positive for Ki-67 immunostaining. The mechanism remains uncertain as to why cancer cells are distributed in a rim fashion in this case. Retained rim enhancement, however, may indicate aggressiveness in mucinous breast cancer. 

## Conclusions

It is well known that pure mucinous breast cancers generally have favorable biologies such as ER positivity, HER2 negativity, node negativity, and low Ki-67 labeling index. It is unclear why this case showed abundant thick fibrous septa and an aggressive phenotype, despite being pure mucinous breast cancer. In addition, it also is unclear how the scarcity or absence of cancer cells in the central part of the mucinous breast cancer correlates the tumor aggressiveness. Diagnostic physicians, therefore, should note that retained rim enhancement may predict the biological aggressiveness of mucinous breast cancers.
